# Future Microbial Applications for Bioenergy Production: A Perspective

**DOI:** 10.3389/fmicb.2017.00450

**Published:** 2017-03-21

**Authors:** Ravinder Kumar, Pradeep Kumar

**Affiliations:** ^1^Department of Biotechnology, ITS Paramedical CollegeGhaziabad, India; ^2^Department of Forestry, North Eastern Regional Institute of Science and Technology (Deemed University)Nirjuli, India

**Keywords:** biofuels, biofuel cell, bioenergy, renewable energy, environmental impact

The fast receding concentration of fossil fuels and the mounting global demand of energy has necessitated the production of alternate fuels to replace the conventional fossil fuels so as to counter the increased deposition of greenhouse gasses in the atmosphere, which has led to considerable climatic changes. These changes could result in catastrophic repercussions in the near future, including rising temperature and sea levels. Evidently, the utilization of fossil fuels for electricity and heat production and for transportation accounts for 25% and 14% of the total greenhouse gas emissions, respectively (IPCC, 2014). Therefore, nowadays, the production of economically feasible and eco-friendly renewable energy fuels is the world's highest demand that indicates the potential to simultaneously replace the conventional fuels and reduce the environmental concern. The use of versatile microorganisms to generate renewable energy fuels from the biomass and biological wastes can diminish this menacing concern to a large extent. The interest in the production of various biofuels using microorganisms has been steadily increasing in the recent years (Table 1) (Liao et al., 2016), particularly because of the metabolic diversity of different microorganisms that enables the production of biofuels from various substrates. For example, most of the bacteria can easily convert sugars into ethanol, and cellulolytic microbes can utilize plant-driven substrates. Cyanobacteria and microalgae possess the potential to photosynthetically reduce the atmospheric CO_2_ into biofuels, and methanotrophs can use methane to produce methanol (Liao et al., 2016). In addition, some of the bacteria such as *Geobacter sulfurreducens* and *Shewanella oneidensis* exhibit specific “molecular machinery” that helps transfer electrons from microbial outer-membrane to conductive surfaces (Kracke et al., 2015), subsequently, this feature can be deployed in bioelectrochemical devices for biohydrogen and bioelectricity generation. The impending need to address the challenges involved in enabling these microorganisms to become a more feasible option for replacing the conventional fossil fuels has been discussed in this paper with possible future directions.

**Table 1:** **List of microorganisms producing biofuels or the precursors for biofuel production**.

**Microorganism**	**Biofuel**	**Biofuel yield (g L^−1^)**	**References**
*Clostridium acetobutylicum*	Butanol	3	Lütke-Eversloh and Bahl, 2011
*Clostridium thermocellum*	Isobutanol	5.4	Lin et al., 2015
*Escherichia coli*	Butanol	30	Shen et al., 2011
*Escherichia coli*	Ethanol	25	Romero-García et al., 2016
*Saccharomyces cerevisiae*	Fatty acids	0.38	Yu et al., 2016
*Saccharomyces cerevisiae*	Isoprenoid based-biofuel	40	Westfall et al., 2012
*Pseudomonas putida*	Butanol	0.05	Nielsen et al., 2009
*Cryptococcus vishniaccii*	Lipids	7.8	Deeba et al., 2016
*Zymomonas mobilis*	2, 3-butanediol	10	Yang et al., 2016
*Zymomonas mobilis*	Ethanol	–	Kremer et al., 2015
*Caldicellulosiruptor bescii*	Ethanol	0.70	Chung et al., 2014
*Trichoderma reesei*	Ethanol	10	Huang et al., 2014
*Yarrowia lipolytica*	Fatty acids	55	Beopoulos et al., 2009
*Synechococcus* sp.	Limonene	0.04	Davies et al., 2014
*Synechococcus elongates*	1, 3-propanediol	0.28	Hirokawa et al., 2016

## Microbial factories for biofuels

**Figure 1:**
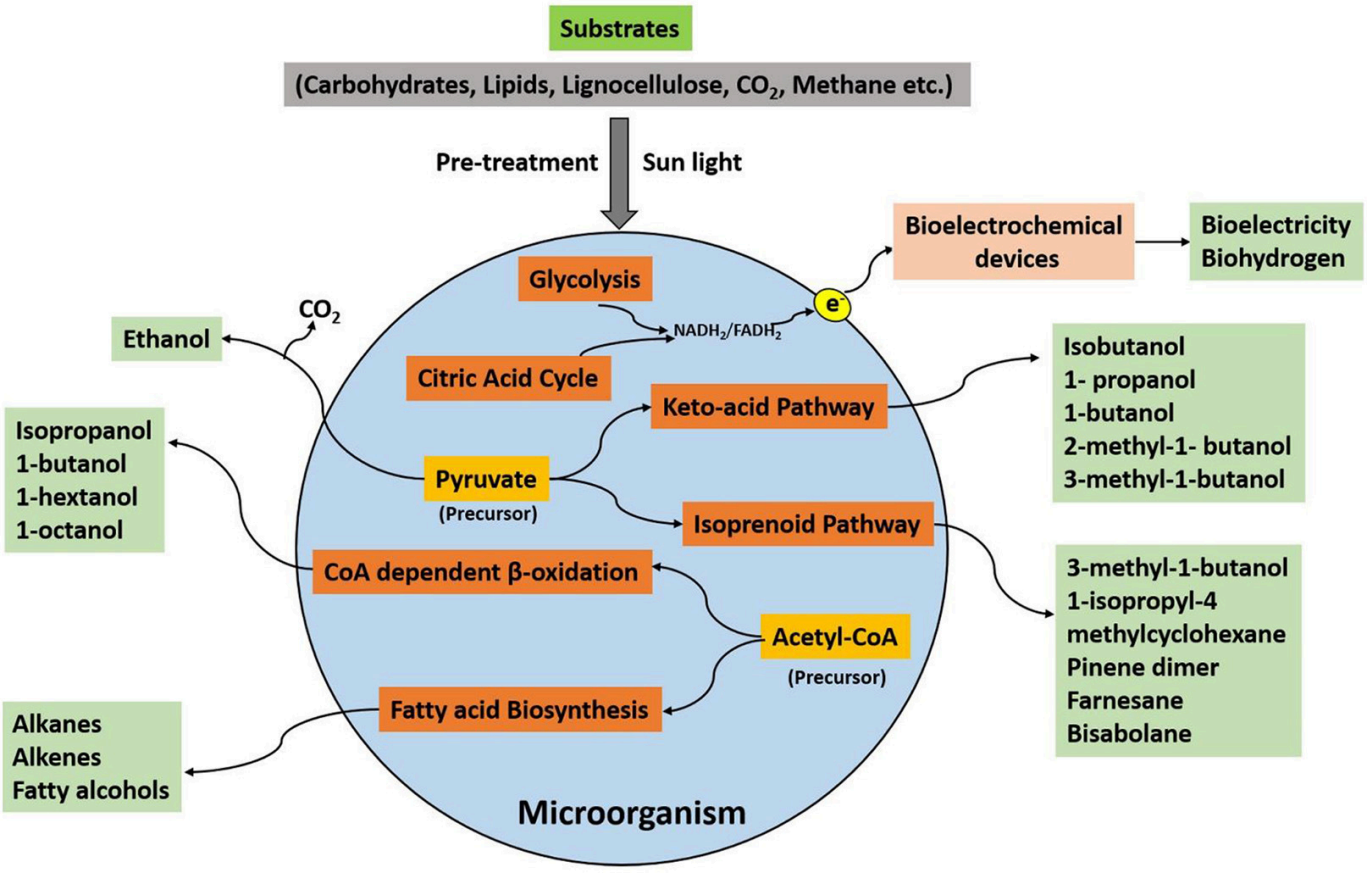
**An overview of microbial metabolic pathways for biofuel production**.

The consumption of organic substrates by a microorganism and its further utilization in the metabolic processes generates useful products, which can be used as a fuel to produce energy. An outline of the microbial pathways for the production of different biofuels has been illustrated in Figure 1. The selection of microbes, substrates, and the production processes are pivotal for biofuel synthesis. The microbial biofuel production, e.g., ethanol from corn, also needs more input of fossil fuel energy as compared to the process involving sugarcane as the substrate (Goldemberg et al., 2008). Therefore, a biofuel with more positive net balance energy is considered suitable for commercialization. The other important concern is the selection of an efficient substrate for microbes. The lignocellulose-containing substrates such as agricultural waste and plant biomass are the most desirable alternatives compared to other types of feedstocks. However, some microorganisms such as *S. cerevisiae* cannot degrade lignocellulose completely into fermentative constituents (Chang et al., 2013).

Plant biomass of lignocellulose can be converted to biofuel through deconstruction to sugars; this process generally starts with a pre-treatment step, followed by enzymatic hydrolysis or by consolidated bioprocessing (which combines the two processes in one reactor) (Mosier et al., 2005; Kumar et al., 2009). This cellulolytic hyphal penetration process can be physical, chemical, biological, or a combination of all of these. The penetrated biomass is then hydrolysed by either non-complexed cellulose enzyme cocktails or by a cellulolytic microorganism (Lynd et al., 2002).

The greenhouse gas methane is emitted in less quantity but is more potent than CO_2_ (Yvon-Durocher et al., 2014), and it is produced from landfill or through anaerobic digestion of various organic wastes. Methane is a major component in natural gas, the production of which has undergone a dramatic flow in the past few decades. This highlights an urgent need to search for more efficient carbon source. Low-throughput methane from a landfill or natural gas wells that is otherwise flared can be used directly by methanotrophs to produce fuels, or it can be converted to methanol (CH_3_OH) and, eventually, utilized by methylotrophs for fuel production (Liao et al., 2016). Methanotrophs oxidize methane by first initiating reduction of oxygen atoms to H_2_O_2_ and then transformation of methane to CH_3_OH using methane monooxygenases (MMOs) (Fuerst, 2013). The MMOs are of two types: soluble MMOs (sMMO) and particulate MMOs (pMMO). The cells containing pMMO have demonstrated higher growth capabilities and higher affinity for methane than sMMO-containing cells.

## Metabolic engineering to upscale biofuel production

The microorganisms exhibit a specific metabolic pathway and different types of catalytic enzymes for biofuel production. For example, in *Saccharomyces cerevisiae*, direct decarboxylation of pyruvate leads to the production of ethanol, while in *Escherichia coli*, CoA activates the acyl group during pyruvate decarboxylation and then reduces to ethanol. Metabolic engineering of such pathways could be fruitful in increasing the productivity of biofuels. This approach can be applied in numerous ways for enhancing microbial biofuel production. First, as mentioned earlier, ethanol can be produced by two different pathways in yeast and in *E. coli*. Ethanol production without using CoA is regarded as an efficient route for ethanol production comparatively (Liao et al., 2016). Therefore, this pathway can be expressed in other microbes through genetic engineering techniques for ethanol production. Similarly, the microorganisms lacking the metabolic pathways for a particular biofuel can be injected with the imperative genes or the enzymes extracted from efficient biofuel producing organism, transforming the non-biofuel producing microorganism to a biofuel-producing microbe. This approach could be beneficial to engineer microbes for exploiting various substrates for biofuel production. Second, the competing pathways that drain the products (biofuels) or the precursors (such as pyruvate, acetyl-CoA) or the enzymes that interfere with the biofuel synthesis pathway can be a knockout with the help of metabolic engineering. For example, in *E. coli*, acyl-ACP (acyl carrier protein) inhibits fatty acid biosynthesis pathway (Davis and Cronan, 2001). The overexpression of thioesterase can avoid this inhibition, allowing the synthesis of free fatty acids, which subsequently results in the production of a precursor (acyl-CoA, for fatty alcohol synthesis). Moreover, the catalytic activity of the substrate-specific enzymes and the number of turnovers can be enhanced by manipulating the genetic material of the enzyme using advanced design tools and experimental techniques. In addition, computation-based proteins can be adopted to structure unnatural amino acids to create artificial enzymes of desired functions that can be further utilized in biofuel production. However, the synthesis of an artificial metabolic pathway could be challenging, requiring advanced effective tools to control the proteins and the mRNA levels for the proper functioning of an artificial pathway.

## Microbial factories in bioelectrochemical devices for bioelectricity and biohydrogen production

In recent years, bioelectrochemical cells (BEC) have gained a significant interest in generating bioenergy from organic biomass and wastewaters. Especially, microbial fuel cells (MFCs) and microbial electrolysis cells (MECs) have been extensively exploited for bioelectricity and biohydrogen production (Logan et al., 2015; Dai et al., 2016). From a biological perspective, both the types of fuel cells function on the similar principle; therefore, common microorganisms can be deployed in these fuel cells for bioenergy production. The unique characteristic of these microorganisms (generally referred to as exoelectrogens or electricigens) in BEC is the exhibition of a specific “molecular machinery” that helps transfer the electrons from microbial outer-membrane to the conductive surfaces (Kracke et al., 2015). Subsequently, the electrons can be used to generate electricity and hydrogen. However, the energy outputs from MFCs and MECs are insufficient for real-world applications, and, currently, not feasible for commercialization. Theoretically, an MFC can produce a maximum voltage of 1.2 V, and the optimum hydrogen production yield in MEC would be 3.4 mol H_2_/mol-acetate (Logan et al., 2015). The real challenge to implement the BEC on a large-scale would be the high cost incurred per unit of energy produced in the system. Moreover, an efficient and suitable BEC design that plays an indispensable role in energy output is required to upscale the BECs. The BECs are in their infancy age, which leaves much scope for exploration of its relative concerns in the future toward improving the bioenergy production. A better understanding of the microbial pathways that are pivotal in BEC's performances such as electroactive biofilm formation, electron transfer mechanisms, and their further manipulations can help improve the energy output from these systems.

The BECs present a substantial platform to explore microbe–metal surface interactions and the microbial physiology to understand the different processes such as biofilm formation and the electron transfer mechanisms between bacteria and electrode surfaces as well as between bacteria. The exoelectrogens exhibit specific redox proteins/molecules that help the transfer of electrons from the microbial outer-membrane to the electrode surface (Kracke et al., 2015). The production of such proteins or the secretion of redox molecules can be enhanced in the exoelectrogens; consequently, more electrons could be transferred at a faster rate. This objective can be achieved by inserting the genes encoding these redox proteins or molecules into the exoelectrogen's genetic material considering that genetic engineering techniques promise great feasibility. Therefore, such genetically engineered exoelectrogens that possess the ability to produce abundant redox proteins can be employed in the BECs to increase the energy output. Similarly, the genes of the biosynthetic pathway for redox proteins can be expressed in non-exoelectrogens to increase the availability of versatile microorganisms in order to exploit the usefulness of numerous substrates for bioenergy production. Moreover, this approach would be extremely effective in terms of decreasing the start-up time and toward improving the performance of BECs. Hence, the use of genetically engineered exoelectrogens can be an advantageous tool in scaling-up the technology.

## Conclusions and future outlook

The most challenging hurdle of producing biofuels using “microbial factories” is to generate a large amount of fuel on a comparatively lower budget and greater efficiency as compared to the conventional fossil fuels. In other words, for replacing petrol with bioethanol, the latter should be cheaper, which could be a highly challenging task in terms of meeting the daily requirement (quantity). For example, in USA, approximately 19 million barrels of petrol is consumed per day; generating this amount on the industrial scale could be an arduous task. Therefore, to increase the acceptability of microbial biofuel, its productivity should be prioritized in the future.

## Author contributions

RK write the manuscript. PK help in writing, edit and finalize the manuscript.

### Conflict of interest statement

The authors declare that the research was conducted in the absence of any commercial or financial relationships that could be construed as a potential conflict of interest.
